# Retraction: LncRNA CHRFinduced miR-489 loss promotes metastasis of colorectal cancer via TWIST1/EMT signaling pathway

**DOI:** 10.18632/oncotarget.28868

**Published:** 2026-04-24

**Authors:** Youmao Tao, Tao Han, Tao Zhang, Chong Ma, Caixia Sun

**Affiliations:** ^1^Department of Gastrointestinal Colorectal and Anal Surgery, China-Japan Union Hospital of Jilin University, Changchun 130033, China

**This article has been retracted:** Oncotarget’s Scientific Integrity office has completed its investigation of this paper. It has been determined that the article contains multiple instances of image duplication and overlap. These issues are both internal to the paper—specifically, the second invasion image in Figure 7C overlaps with the third image—and external, involving other published works. Five earlier published articles, all studying different types of tumors, share a significant number of duplicated and overlapping images with this paper:

- Figure 2: The wound healing assay images in Figures 2B and 2E are duplicates of Figures 2F and 2G from 2014 paper [1]. Additionally, the invasion assay images in Figure 2F overlap with images in Figure 3D of [2], Figure 4D of [3] and Figure 3C of [4].
- Figure 3: The H&E staining images in Figure 3 are duplicates of the liver panels in Figure 4B from an earlier published unrelated paper [5].
- Figure 4: The morphology and immunofluorescence staining images in Figure 4A and 4B are duplicates of images in Figure 1A and 1B, and the western blot images are duplicates of those in Figures 2C and 3B of 2014 article [6].
- Figure 5: The western blots for TWIST1 and GAPDH in Figure 5D were previously published as E-cadherin and b-actin in Figure 3E of [6].
- Figure 7: The FOXO1 western blot in Figure 7A was found as the TWST1 blot in Figure 6B of 2014 article [7]. The wound healing assy images in Figure 7B were previously published in Figure 5B of [1], and the transwell assay images in Figure 7C were previously published in Figure 6B of [4].
- Figure 9: The TWIST1 western blots in Figures 9A and 9C have been reused as p-STAT3 in Figure 6 of [3], which shares two authors with this article and was recently retracted. Additionally, the bright field micrographs in Figure 9B and 9D (colon cancer cells) were previously published as epithelial cancer cells in Figures 3A and 3D of [6].

Oncotarget contacted the authors, and while they initially promised to provide an explanation and the original data, we have not heard from them since, despite our repeated attempts.

Taking into account all of the identified inconsistencies, the Editorial decision has been made to retract this article. The authors have been informed of this decision but have not responded.

Original article: Oncotarget. 2017; 8:36410–36422. 36410-36422. https://doi.org/10.18632/oncotarget.16850
